# Supplementary material for: Rice OsHSFA3 Gene Improves Drought Tolerance by Modulating Polyamine Biosynthesis Depending on Abscisic Acid and ROS Levels
Source: Int J Mol Sci. 2020 Mar 9;21(5):1857. doi: 10.3390/ijms21051857 (PMC7084839; doi:10.3390/ijms21051857)
Supplement: Supplementary file 1 [file ijms-21-01857-s001.pdf]

Supplementary Material

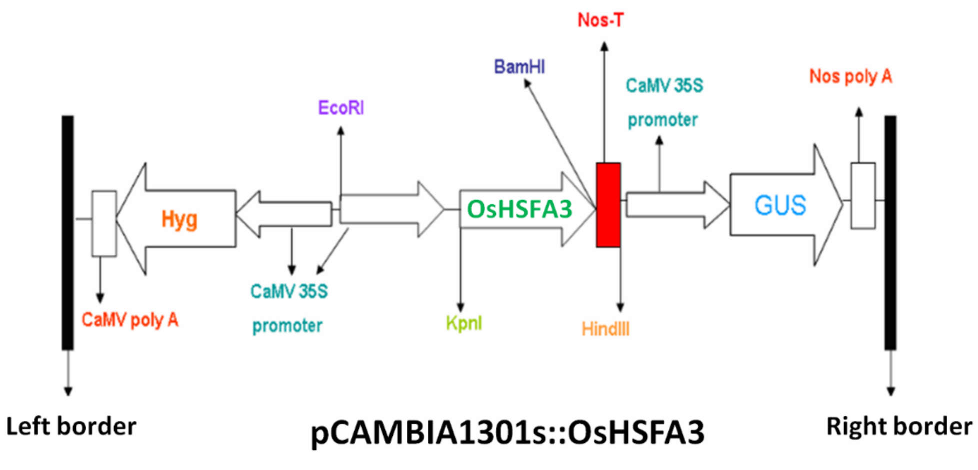

Figure S1. Map of the vector used for the transformation of *Arabidopsis* thaliana.

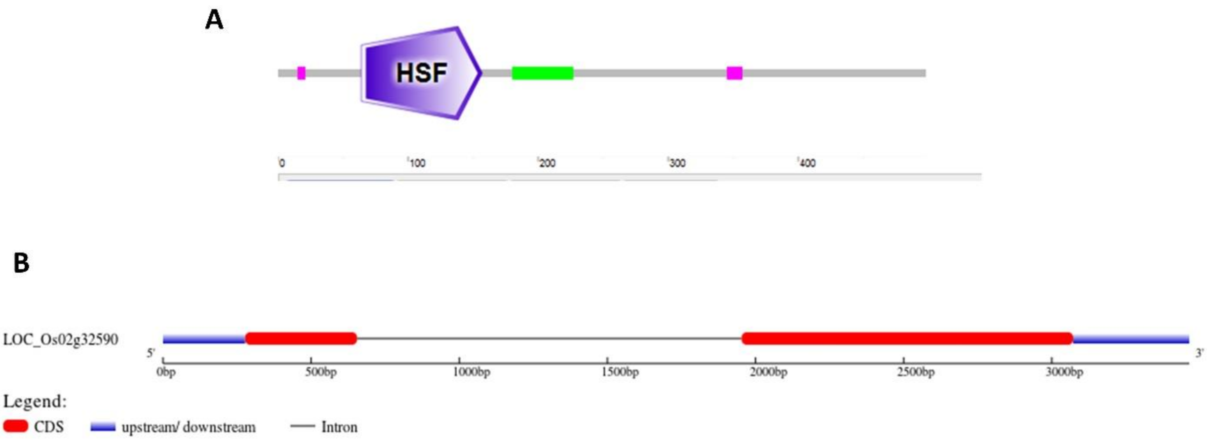

Figure S2. Conserved domains and gene structure of *OsHSFA3*. (**A**) Conserved domains in OsHSFA3 protein. (**B**) Gene structure displaying intron and exons in *OsHSFA3* gene.

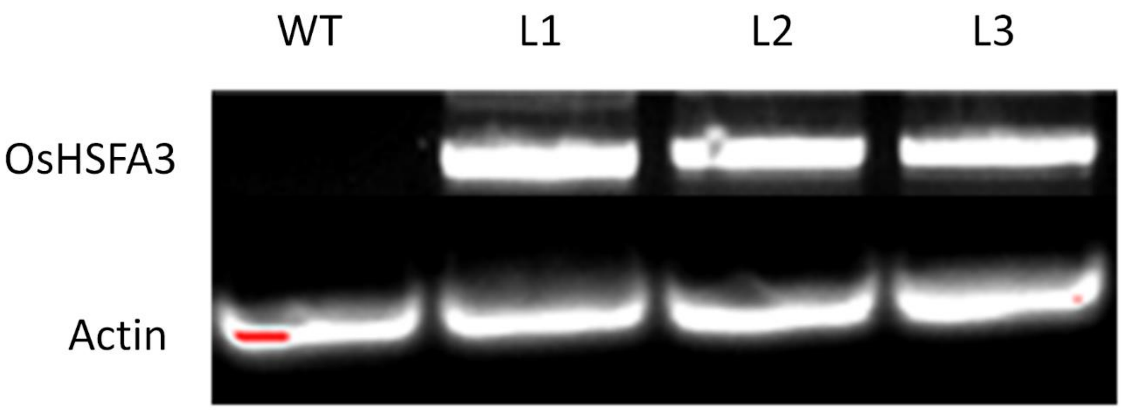

Figure S3. Identification of positive *Arabidopsis* over-expressing lines. Reverse transcriptase polymerase chain reaction analysis of *OsHSFA3* in the wild type (WT) and transgenic lines (#L1, #L2 and #L3). The *Actin* gene was used as internal control.

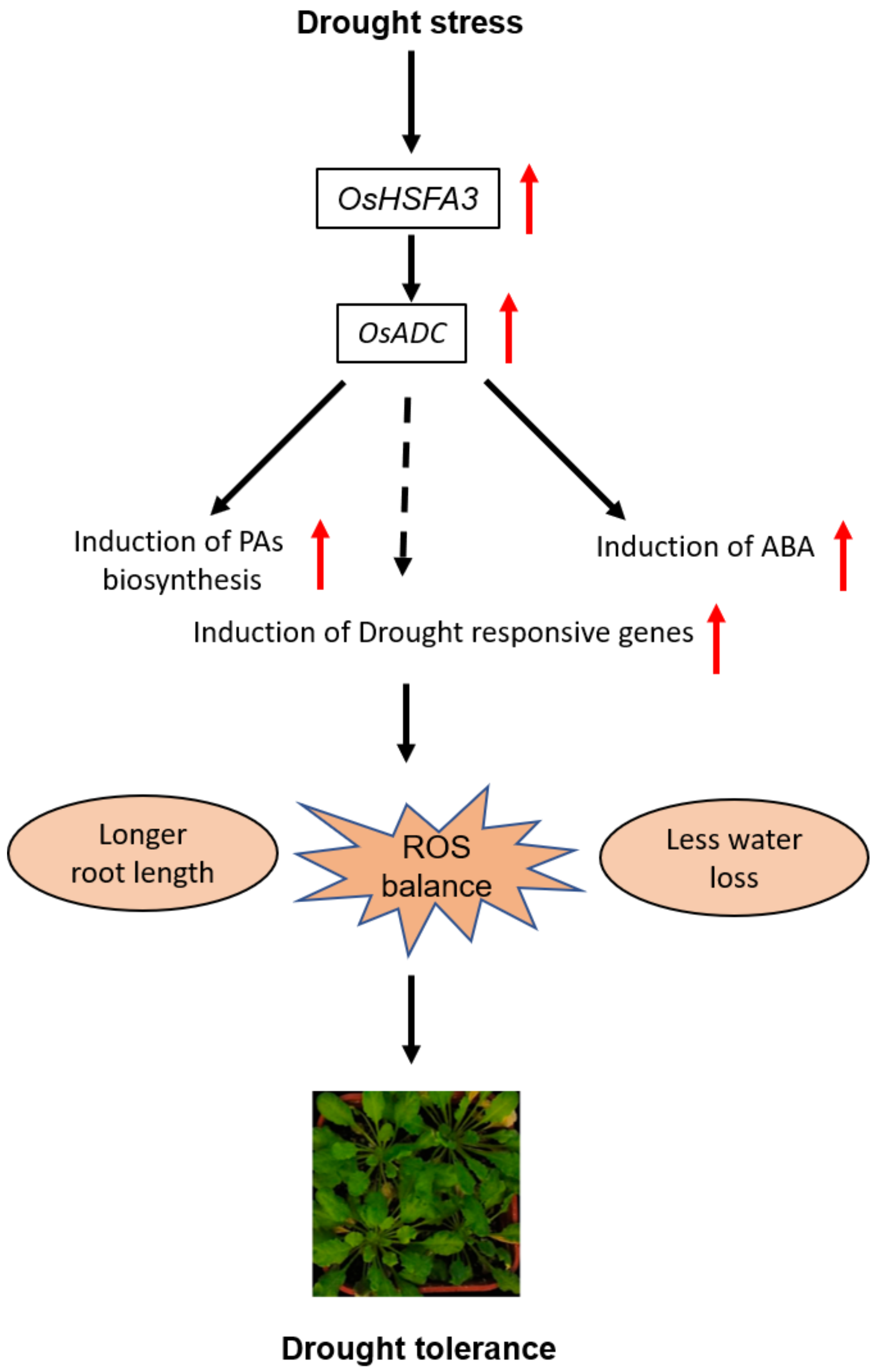

Figure S4. Proposed working model for *OsHSFA3*. Drought stress induces *OsHSFA3* expression which regulates the expression of polyamines (PAs) biosynthesis gene *"OsADC"*. It then stimulates the ABA and PAs biosynthesis and possibly induces expression of stress responsive genes. These physiological changes maintain ROS homeostasis, longer root length and protect water loss under drought stress which collectively leads toward drought tolerance in rice.

Table S1. Primer sequences used in the study.

	Gene Name	Primer Sequence
**1**.	*AtADC1_F*	TATGCTTGAAGGGTACTCTG
	AtADC1_R	TCGTTCCCCTTATCACCAC
**2**.	*AtADC2_F*	ACGGGTCCGAAATCTTCC
	AtADC2_R	CGGGAGTTCCTTGATGAA
**3**.	*AtSPDS1_F*	TCTCTCTCATTTCTCGGAG
	AtSPDS1_R	CAAGAAAGCAACACCATCG
**4**.	*AtSPMS_F*	AATGTTCTTGTTGTTGGTGGAG
	AtSPMS_R	ACTTCTCTCCGCAGGAATGTG
**5**.	*AtActin2_F*	TCCTGCTCGTAGTCAA
	AtActin2_R	CTCCCGCTATGTATGT
**6**.	*OsHSFA3_F*	CAACTCCGCTACCAAG
	OsHSFA3_R	TCCTCCAGAACCCAACT
**7**.	*OsADC_F*	TCCGGGTAGAACGACAGG
	OsADC_R	TCATCAGCGGGAGGCA
**8**.	*OsActin-1_F*	CTTCATAGGAATGGAAGCTGCGGGTA
	OsActin-1 gene_R	CGACCACCTTGATCTTCATGCTGCTA
